# Maskerading Bundle Branch Block Versus Standard Bifascicular Block: A Prognostic Analysis Results Through Electrophysiological Study

**DOI:** 10.1111/anec.70235

**Published:** 2026-09-12

**Authors:** Afonso Luiz Tavares de Albuquerque, Caroline de Paula Coutinho Vaz de Lira, José Nilo de Carvalho Neto, Rodrigo Rufino Pereira Silva, Iremar Salviano de Macedo Neto, Dário Celestino Sobral Filho

**Affiliations:** ^1^ Division of Cardiology, Pronto Socorro Cardiológico de Pernambuco Prof. Luiz Tavares (PROCAPE) Universidade de Pernambuco (UPE) Recife Brazil

**Keywords:** electrophysiological study, heart block, masquerading branch block

## Abstract

**Background:**

Maskerading bundle branch block (MBBB) is a rare variant of standard bifascicular bundle branch block (SBBB), associated with poor prognosis and a higher need for pacemaker implantation. Most available data are limited to case reports and reviews.

**Methods:**

In this prospective cross‐sectional study, we evaluated 27 patients with MBBB (Group I) and 18 with SBBB (Group II), all undergoing echocardiography, 24‐h Holter monitoring, and invasive electrophysiological study (EPS) after recruitment.

**Results:**

Group I patients had worse functional status (NYHA III–IV), larger ventricular diameters, and lower ejection fraction. The HV interval was significantly longer in Group I (66.3 ± 18.3 vs. 52.9 ± 8.1 ms; *p* = 0.006). Pacemaker implantation was required in 48% of Group I patients versus none in Group II (*p* = 0.001). Persistent atrial fibrillation was also more frequent in Group I (39.5% vs. 16.6%; *p* = 0.018).

**Conclusion:**

MBBB is associated with more severe myocardial and conduction system impairment compared to SBBB, supporting its role as a marker of worse prognosis.

## Graphical abstract

Patients with masked bundle branch block (MBBB) exhibited significantly longer HV intervals than those with standard bifascicular block (66.3 vs. 52.9 ms; *p* = 0.006), suggesting more advanced His–Purkinje conduction disease. During follow‐up, MBBB was associated with higher rates of atrial fibrillation or flutter (39.5% vs. 12.0%; *p* = 0.018) and permanent pacemaker implantation (29.5% vs. 0%; *p* = 0.001). These findings indicate that MBBB identifies a subgroup with greater electrophysiological impairment and increased risk of clinically relevant arrhythmic outcomes.

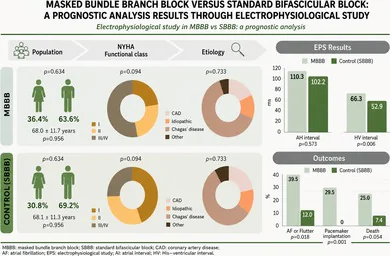

**Abbreviations**
cSNRT: corrected sinus node recovery time
ECG: electrocardiogram
EPS: electrophysiological study
LAFB: left anterior fascicular block
LBBB: left bundle branch block
MBBB: masquerading bundle branch block
RBBB: right bundle branch block
SBBB: standard bifascicular bundle branch block
TTE: transthoracic echocardiography

## 1 Introduction

Masquerading bundle branch block (MBBB), or occult left bundle branch block (LBBB), is a rare form of bifascicular block (Surawicz et al. 2009; Bayés de Luna et al. 1988). It was first described in 1954 by Richman et al. (Richman and Wolff 1954) and was subsequently recognized by other authors, including Lepschkin (Lepeschkin 1964) and Rosenbaum (Rosenbaum et al. 1968); it is characterized by a pattern of right bundle branch block (RBBB) in the horizontal plane (V1 lead) and morphology of LBBB in limb leads with qR waves or absent S waves in lead –aVR or D1, with D1 and aVL leads with QRS left axis deviation in frontal plane leads (Schamroth and Dekock 1975) (Supporting Information—Figures [Link], [Link]).

There are two described patterns of MBBB, namely, the standard type and the precordial type. In the first commonly described standard type, the precordial leads V1–V6 demonstrate the RBBB pattern, whereas the limb leads demonstrate an LBBB pattern. This pattern could be the result of a high degree of left anterior hemiblock or concomitant left anterior posterior block. In the precordial type, there is an LBBB pattern in leads V4–V6 with the absence of S waves and an RBBB pattern in precordial leads V1–V3 (Bayés de Luna et al. 1988).

The presence of a MBBB pattern on the electrocardiogram (ECG) could indicate severe fibrotic degeneration of the left bundle pathways and an elevated risk of progression to atrioventricular dissociation. It is associated with high mortality (41.4%) and pacemaker insertion (38.9%), with combined endpoints of 80.2% over 4 years (Souza et al. 2015), despite most studies on this topic being case reports and reviews.

The most common etiologies of MBBB include ischemic and hypertensive cardiomyopathies (Surawicz et al. 2009). In Brazil, chronic Chagas cardiomyopathy is the most common etiology of MBBB (Souza et al. 2015). It was hypothesized that this type of bifascicular block is related to a higher degree of conduction abnormalities, and the purpose of this study was to assess the extension of the damage to the conduction system through an electrophysiological study (EPS) of patients with MBBB and to compare the latter with patients with standard bifascicular block.

## 2 Methods

This prospective cross‐sectional study with group comparison sought to evaluate the degree of functional changes through the analysis of left ventricular ejection fraction, analyze the conduction system through EPS with His bundle electrogram baseline measurements, and compare patients with MBBB (Group I) and patients with SBBB (Group II) to the incidence of a pacemaker implantation during follow‐up.

The study was approved by the Research Ethics Committee of our institution. Patients with MBBB and patients with RBBB + left anterior fascicular block (LAFB) were invited to participate in the study and were informed about the risks of electrophysiological examination. The patients underwent clinical and electrocardiographic evaluation by a team of professionals. The examinations included two‐dimensional echocardiogram with Doppler, 24‐h Holter monitoring, and an EPS of the conduction system. Routine laboratory tests, serological tests for Chagas' disease, and coronary angiography were performed in some cases to determine the etiology.

Patients were randomly enrolled in the study from December 2014 to August 2016 according to the day of hospitalization and the day the information on the above electrocardiographic changes was provided to the medical team. Patient groups were formed as previously described. The exclusion criteria considering the study objectives and the local ethics committee recommendations were: Patients aged < 18 years, those with signs of hemodynamic instability due to clinically assessed low cardiac output, pregnant women, and those with permanent total atrioventricular block (due to the predicted findings at EPS).

The EPS was performed in the hemodynamics unit under local anesthesia and minimum sedation, according to the conventional techniques (Rahimtoola et al. 1987). The procedure was performed by a specialist who was blind to patient allocation, and the measurements of the His bundle electrogram intervals were made directly on the polygraph recordings. Catheters were placed inside the heart by puncture of the right femoral vein and were positioned at a high location in the right atrium and at the His bundle region while recording the electrogram.

The AH and HV intervals and the corrected sinus node recovery time (cSNRT) were measured in cases in which sinus rhythm was present. Only the HV interval was measured in the presence of atrial fibrillation. Programmed ventricular stimulation to evaluate ventricular stability was not performed by recommendation of the ethics committee because the patients' condition was severe and they were at a high risk of developing malignant arrhythmia.

In the descriptive statistical analysis, the categorical variables were expressed as frequencies and percentages. The variables with a normal distribution were expressed as means and standard deviations. In the comparative analysis, the chi‐square test or Fisher's exact test was used to compare the proportions, and the Student's *t*‐test was used to compare the means. The level of significance was set at 0.05. The analyses were performed using the Stata software version 12.1 (StataCorp, College Station, Texas, USA). The complete analysis of all patients can be found in the Supporting Information—Table S4.

## 3 Results

Seventy patients were evaluated; Forty‐five consecutive patients with an MBBB pattern in the ECG were included and the majority exhibited a conventional pattern; 26 patients had SBBB pattern (Supporting Information—Table S1). The results for gender, mean age, and etiology were similar between the groups. The mean age was 68 (±11.7) years in Group I and 68.1 (±11.3) years in Group II. The most common cardiomyopathy etiology was chronic Chagas disease, followed by ischemic cardiomyopathy. The electrocardiographic behavior was similar between the groups regarding atrioventricular conduction (PR interval), QRS width, and electrical axis, with a higher rate of atrial fibrillation rhythm (31%) in Group I than in Group II (12%) with statistical significance (*p*: 0.018).

The patients in Group I belonged predominantly to functional classes III and IV and had higher left chamber diameters and worsened ejection fraction on the Doppler echocardiogram (Supporting Information—Table S2). At the follow‐up, 29.5% of the patients in Group I required pacemaker implantation and none in Group II (*p*: 0.001), while 11 patients died in Group I (25%) and only 2 in Group II (7.4%) with *p*: 0.054.

The EPS was performed in 45 patients (64%) (Supporting Information—Table S3), which included 27 patients in Group I and 18 patients in Group II. Seventeen patients in Group I and eight patients in Group II did not undergo the examination because of contraindications, including severe heart failure, or did not consent to undergo the procedure.

There was no significant difference in the mean AH interval (in ms) between the two groups (110 ± 54 vs. 102 ± 24.9; *p* = 0.573). Thirteen patients in Group I presented atrial fibrillation rhythm. Three patients in Group I had HV interval values > 100 ms and one patient had a value of 110 ms. The cSNRT was abnormal (> 500 ms) in five patients, and these patients required permanent ventricular stimulation. The latter patient had been hospitalized because of syncope. Complete patients' data such as their baseline characteristics (Clinical parameters, variables, echocardiogram measures and EPS‐HV values) are described at Table S4 in Supporting Information.

## 4 Discussion

Most studies on MBBB are case reports or reviews (Elizari et al. 2007; Shah and Gandhi 1986; Kaimoto et al. 2013; Scheidt and Killip 1972; Kukla et al. 2014; Schneider et al. 1980), although MBBB is known since 1954. Wilson et al. (Wilson et al. 1934) first described this unusual form of intraventricular conduction disorder. The mechanisms underlying MBBB are not fully understood. Despite the pattern of LBBB and RBBB, total block is not present but rather bundle branch conduction is delayed. The absence of the S wave in DI and aVL in MBBB has been explained by the delayed depolarization of the lateral wall of the LV, which occurs at the same time or after RV depolarization, masquerading in the frontal plane the manifestations of the terminal delay of the typical RBBB (Rosenbaum et al. 1968).

The histopathological evaluation performed in two hearts (Unger et al. 1958) indicated severe structural impairment of the myocardium and conduction abnormalities bilaterally, which probably explains the severity of MBBB.

In 1988, Bayés De Luna (Surawicz et al. 2009) considered MBBB a rare condition after reviewing 100,000 electrocardiograms and finding only 16 cases (mostly male patients with mean age of 70 ± 9 years). Most patients (68%) in this case series had ischemic cardiomyopathy. Cardiomegaly on chest X‐ray was observed in 13 patients (81%) and 68% of patients belonged to the New York Heart Association functional classes III and IV. During a follow‐up period of 39.1 ± 32 months, 10 patients (62%) required a pacemaker implant in the first year, the majority (nine patients) for third‐degree AVB. Invasive EPS was not performed.

Subsequently, in 1997, Barrado et al. (Gómez Barrado et al. 1997) published a series of 22 cases, with a follow‐up period of 3 years, and observed a much higher rate than that described by Bayés De Luna. The clinical characteristics of these patients were like those of the patients in the study by Bayés De Luna; however, 15 patients needed a permanent pacemaker implant, 13 of them (57%) for total AVB or for increased degree of AVB; the indication of four patients was confirmed by the EPS because of the presence of infra‐His‐ian conduction delay.

In a case report study, Kowey et al. (1989) examined a 69‐year‐old male patient who presented with presyncope. The ECG showed a MBBB pattern and first‐degree AVB. He exhibited an enlarged heart on chest X‐ray and mild changes in LV function on echocardiogram. The results of the EPS showed a HV interval of approximately 120–140 ms, which confirmed severe bilateral injury of the conduction system.

Souza et al. (2015) selected 25 cases among 800,000 electrocardiograms stored in the system of a telemedicine service. The mean age of these patients was higher, and there was a higher incidence of atrial fibrillation rhythm (28%) and atrial flutter (4%). Of the 25 selected patients, 15 were followed up, and, of these, four (26.6%) required a pacemaker implant, seven (46.6%) died, and nine (60%) had combined outcomes. EPS was not performed in this series.

The present case series study is the first to compare a MBBB group and a control group of patients with standard bifascicular bundle branch block (RBBB+LAFB). Forty‐four patients with MBBB and 26 control patients were assessed. There was a similar distribution of patient age, gender, place of origin, and etiology between the groups.

In this sample, there was a predominance of patients with Chagas' disease (66.7%) in both groups, in contrast to previous studies in which ischemic disease predominated. The high rate of persistent fibrillation and atrial flutter (31.7%) may be because these patients had a worse ejection fraction and cavity diameters of the LV. Despite the shorter follow‐up period (18 months), several patients with MBBB required pacemaker implants because of bradyarrhythmia, which is in line with the results of previous studies.

An EPS (Supporting Information—Figure S4) was performed in 27 patients in Group I and 18 patients in Group II; the mean AH interval was similar between the two groups. However, the mean HV interval was increased (66.3%) in Group I and was significantly different from that in Group II. There was a significant increase in three patients (> 100 ms), indicating a greater injury of the His–Purkinje system and a greater probability of trifascicular involvement of the conduction system; this may explain the higher risk of progression to total AVB. Therefore, the presence of MBBB in this sample corresponded to disease severity, especially in patients with syncope during disease progression.

In relation to the population analyzed in the study, those with Chagas disease deserves to be highlighted. Given the unique nature of their condition and the limitations of current studies, we understand that multiple mechanisms may be responsible for the development of cardiac changes in individuals infected with *T. cruzi* that include specific characteristics of the host and parasite, which require further investigation. It is not entirely clear which structural changes directly lead to the different presentations of cardiac arrhythmias that occur in this population. One most likely hypothesis is that a high fibrotic load resulting from local inflammatory changes could affect directly the His‐Purkinje system. Magnetic resonance imaging exams could give us more information in correlation to EPS.

A recent meta‐analysis (Rojas et al. 2018) involving 34,023 patients showed that among specific ECG abnormalities in this population, there is a prevalence of RBBB (OR = 4.60; 95% CI = 2.97–7.11), LAFB (OR = 1.60; 95% CI = 1.21–2.13), RBBB+LAFB (OR = 3.34; 95% CI = 1.76–6.35), and first‐degree atrioventricular block (A‐V B) (OR = 1.71; 95% CI = 1.25–2.33), which suggests the magnitude of the problem.

Considering the observational nature of the study and sample size, and despite the confirmation of the poor outcome of patients with MBBB, the routine evaluation of this group with bilateral branch block needs to be better assessed; and in the presence of syncope or presyncope, pacemaker implantation should be considered. Moreover, whether the use of a resynchronization pacemaker implant changes the natural course of the underlying disease needs to be clarified.

### 4.1 Limitations of the Study

Some patients refused invasive evaluation; this may have influenced the results by reducing the number of eligible individuals during the study. The higher incidence of persistent atrial fibrillation in the MBBB group made it impossible to measure the HA interval and cSNRT, compromising the analysis of the sinus node and atrioventricular node. A important limitation was the failure to perform magnetic resonance imaging (due to logistic limitations) in this patients to assess the fibrosis degree, which could probably correlate with the EPS findings, especially in those with Chagas disease, which we believe have highest fibrotic burden given the inflammatory nature of this disease and its poor long‐term evolution. Left ventricular ejection fraction was assessed using the Teichholz method because this measurement was consistently available in the retrospective dataset. Although this approach allowed standardized analysis across the study population, it may be less accurate than the biplane Simpson method, particularly in patients with regional wall‐motion abnormalities or altered left ventricular geometry.

## 5 Conclusions

The present study is the first to include a control group with bifascicular bundle branch block. Our results and those from previous studies demonstrated that MBBB was associated with higher severity because these patients presented significant anatomical and functional changes of the LV.

A pacemaker needs to be implanted during disease progression because of diffuse bilateral injury of the conduction system, as demonstrated by the increased mean HV interval. The higher prevalence of atrial fibrillation and the greater tendency to die in this group is probably due to greater structural involvement of the heart.

Therefore, patients with MBBB should be assessed more frequently and the implant of a pacemaker should be seriously considered in cases in which these patients present symptoms of low cardiac output, including presyncope and syncope.

### 5.1 Highlights

Masquerading bundle branch block is an easily overlooked pattern on the ECG that indicates severe disease of the atrioventricular nodal conduction pathway. This cases, often caused by coronary artery disease, infiltrative diseases, a series of different cardiomyopathies and idiopathic degeneration of the atrioventricular nodal conduction pathways, could be easily missed as it needs a detailed interpretation of the ECG in addition to the clinical presentation of the patient.

This study demonstrates that the MBBB pattern is correlated with poor clinical status, greater disease progression, worsened LVEF, and increased risk of arrhythmias and need of pacemaker. The clinical perspectives must be targeted to the development of greater new studies that focus on understanding how the clinician could better manage these patients.

Given its rarity, this clinical entity risks misdiagnosis and inappropriate management. This work highlights two challenges for clinicians: To recognize the rarely described masquerading bundle branch block pattern and correlates this with the clinically results and prognosis. Isn't clear if these patients could have additional benefits with an upgrade of their devices to a resynchronizer.

## Author Contributions

Afonso Luiz Tavares de Albuquerque and Dário Celestino Sobral Filho contributed to the study conception and design, methodology, supervision, and critical revision of the manuscript. Caroline de Paula Coutinho Vaz de Lira, José Nilo de Carvalho Neto, Rodrigo Rufino Pereira Silva, and Iremar Salviano de Macedo Neto contributed to data collection, data curation, clinical investigation, and interpretation of the findings. Afonso Luiz Tavares de Albuquerque and Rodrigo Rufino Pereira Silva drafted the initial version of the manuscript. All authors contributed to the review and editing of the manuscript, approved the final version, and agree to be accountable for all aspects of the work.

## Funding

The authors have nothing to report.

## Disclosure

The authors have nothing to report.

## Conflicts of Interest

The authors declare no conflicts of interest.

## Supporting information


**Figure S1:** Masquerading bifascicular block; Note the absence of S wave in lead I and atrial fibrillation. Data source: Electronic health record (EHR) data.

**Figure S2:** Masquerading bifascicular block without S wave in lead I. Data source: Electronic health record (EHR) data.

**Figure S3:** Classic bifascicular block, RBBB with S wave in lead I plus LAFB. Data source: Electronic health record (EHR) data.

**Figure S4:** His bundle electrogram in patient with Masquerading bundle branch block. Increased HV interval (82 ms). Data source: Electronic health record (EHR) data.

**Table S1:** Comparison of the distribution of clinical, etiological, and electrocardiographic variables and the need for permanent pacemaker implant.

**Table S2:** Comparison of the means of the ETT variables between the MBBB and RBBB plus LAFB groups.

**Table S3:** Characteristics of the invasive electrophysiological study.

**Table S4:** Baseline characteristics of the studied patients (Clinical parameters, variables, echocardiogram measures and EPS‐HV values).

## Data Availability

The authors confirm that the data supporting the findings of this study are available within the article [and/or] its Supporting Information. Data available within the article or its Supporting Information.

## References

[anec70235-bib-0001] Bayés de Luna, A. , P. Torner , R. Oter , et al. 1988. “Study of the Evolution of Masked Bifascicular Block.” Pacing and Clinical Electrophysiology 11, no. 11 Pt 1: 1517–1521. 10.1111/j.1540-8159.1988.tb06267.x. 2462233

[anec70235-bib-0002] Elizari, M. V. , R. S. Acunzo , and M. Ferreiro . 2007. “Hemiblocks Revisited.” Circulation 115, no. 9: 1154–1163. 10.1161/CIRCULATIONAHA.106.637389. 17339573

[anec70235-bib-0003] Gómez Barrado, J. J. , S. Turégano Albarrán , J. C. García Rubira , et al. 1997. “Clinical and Electrocardiographic Characteristics of Masquerading Bifascicular Block.” Revista Española de Cardiología 50: 92–97. 10.1016/s0300-8932(97)73185-3. 9092008

[anec70235-bib-0004] Kaimoto, S. , T. Kawasaki , T. Taniguchi , S. Kawasaki , T. Kamitani , and H. Sugihara . 2013. “Masquerading Bundle Branch Block as a Marker of Poor Prognosis.” Journal of Cardiology Cases 8, no. 1: e57–e59. 10.1016/j.jccase.2013.04.005. 30546743 PMC6281492

[anec70235-bib-0005] Kowey, P. R. , M. Koslow , R. A. Marinchak , and T. E. D. Friehling . 1989. “Masquerading Bundle Branch Block. Electrophysiological Correlation.” Journal of Electrophysiology 3: 156–159. 10.1111/j.1540-8167.1989.tb01543.x.

[anec70235-bib-0006] Kukla, P. , A. Baranchuk , M. Jastrzębski , and L. Bryniarski . 2014. “Zamaskowany blok odnogi pęczka Hisa [Masquerading bundle branch block].” Kardiologia Polska 72, no. 1: 67–69. Polish. 10.5603/KP.2014.0006. 24469750

[anec70235-bib-0007] Lepeschkin, E. 1964. “The Electrocardiographic Diagnosis of Bilateral Bundle Branch Block in Relation to Heart Block.” Progress in Cardiovascular Diseases 6: 445–471. 10.1016/s0033-0620(64)80002-5. 14153649

[anec70235-bib-0008] Rahimtoola, S. H. , D. P. Zipes , M. Akhtar , et al. 1987. “Consensus Statement of the Conference on the State of the Art of Electrophysiologic Testing in the Diagnosis and Treatment of Patients With Cardiac Arrhythmias.” Circulation 75, no. 4 Pt 2: III3–III11. 3829359

[anec70235-bib-0009] Richman, J. L. , and L. Wolff . 1954. “Left Bundle Branch Block Masquerading as Right Bundle Branch Block.” American Heart Journal 47, no. 3: 383–393. 10.1016/0002-8703(54)90295-1. 13124253

[anec70235-bib-0010] Rojas, L. Z. , M. Glisic , L. Pletsch‐Borba , et al. 2018. “Electrocardiographic Abnormalities in Chagas Disease in the General Population: A Systematic Review and Meta‐Analysis.” PLoS Neglected Tropical Diseases 12, no. 6: e0006567. 10.1371/journal.pntd.0006567. 29897909 PMC5999094

[anec70235-bib-0011] Rosenbaum, M. B. , M. V. Elizari , and J. O. Lazzari . 1968. Los Hemibloqueos. Paidos.

[anec70235-bib-0012] Schamroth, L. , and J. Dekock . 1975. “The Concept of ‘Masquerading’ Bundle‐Branch Block.” South African Medical Journal 49: 399–400. 1145361

[anec70235-bib-0013] Scheidt, S. , and T. Killip . 1972. “Bundle‐Branch Block Complicating Acute Myocardial Infarction.” Journal of the American Medical Association 222, no. 8: 919–924. 10.1001/jama.1972.03210080011003. 4678959

[anec70235-bib-0014] Schneider, J. F. , H. E. Thomas , B. E. Kreger , P. M. McNamara , P. Sorlie , and W. B. Kannel . 1980. “Newly Acquired Right Bundle‐Branch Block: The Framingham Study.” Annals of Internal Medicine 92, no. 1: 37–44. 10.7326/0003-4819-92-1-37. 7350871

[anec70235-bib-0015] Shah, V. K. , and M. J. Gandhi . 1986. “Masquerading Bundle Branch Block.” Journal of the Association of Physicians of India 34: 871–872. 3584044

[anec70235-bib-0016] Souza, T. G. S. , R. L. Almeida , G. P. Targueta , et al. 2015. “Masquerading Bundle Branch Block: An Electrocardiographic Marker of Poor Prognosis.” Circulation 132: A14845. 10.1161/circ.132.suppl_3.14845.

[anec70235-bib-0017] Surawicz, B. , R. Childers , B. J. Deal , et al. 2009. “AHA/ACCF/HRS Recommendations for the Standardization and Interpretation of the Electrocardiogram: Part III: Intraventricular Conduction Disturbances: A Scientific Statement From the American Heart Association Electrocardiography and Arrhythmias Committee, Council on Clinical Cardiology; the American College of Cardiology Foundation; and the Heart Rhythm Society. Endorsed by the International Society for Computerized Electrocardiology.” Journal of the American College of Cardiology 53, no. 11: 976–981. 10.1016/j.jacc.2008.12.013. 19281930

[anec70235-bib-0018] Unger, P. N. , M. E. Lesser , V. H. Kugel , et al. 1958. “The Concept of Masquerading Bundle‐Branch Block; an Electrocardiographic Correlation.” Circulation 17: 397–409. 10.1161/01.cir.17.3.397. 13511659

[anec70235-bib-0019] Wilson, F. N. , F. D. Johnston , and P. S. Barker . 1934. “Electrocardiogram of Unusual Type in Right Bundle Branch Block.” American Heart Journal 9: 472. 10.1016/S0002-8703(34)90095-8.

