# Supplementary material for: Maskerading Bundle Branch Block Versus Standard Bifascicular Block: A Prognostic Analysis Results Through Electrophysiological Study
Source: Ann Noninvasive Electrocardiol. 2026 Sep 12;31(5):e70235. doi: 10.1111/anec.70235 (PMC13570261; doi:10.1111/anec.70235)
Supplement: Supplementary file 3 [file ANEC-31-e70235-s003.docx]

**Table S2.** Comparison of the means of the ETT variables between the MBBB and RBBB plus LAFB groups.

|  | Group | | | |  |
| --- | --- | --- | --- | --- | --- |
|  | MBBB | | Control | |  |
| Variable | N | Mean (SD) | N | Mean (SD) | P^*^ |
| LA volume index (ml/m^2^) | 40 | 44.8 (8.3) | 23 | 40.1 (7.1) | **0.026** |
| LVSD (mm) | 39 | 53.0 (11.6) | 23 | 43.0 (12.7) | **0.002** |
| LVDD (mm) | 39 | 65.2 (9.4) | 23 | 57.6 (10.9) | **0.005** |
| IVS (mm) | 39 | 8.6 (2.2) | 23 | 9.3 (2.0) | 0.157 |
| PW (mm) | 39 | 8.6 (1.9) | 23 | 9.2 (1.9) | 0.292 |
| LVEF (Teicholz %) | 41 | 37.2 (13.9) | 23 | 51.0 (14.6) | **<0.001** |

**Caption**: ^*^Student’s t-test; MBBB: Masquerading bundle branch block; LA: Left atrium; LVSD: Left ventricular systolic diameter; LVDD: Left ventricular diastolic diameter; IVS: Interventricular septum; PW: Posterior wall; LVEF: Ejection fraction of the LV; SD: Standard deviation.
