# Supplementary material for: Maskerading Bundle Branch Block Versus Standard Bifascicular Block: A Prognostic Analysis Results Through Electrophysiological Study
Source: Ann Noninvasive Electrocardiol. 2026 Sep 12;31(5):e70235. doi: 10.1111/anec.70235 (PMC13570261; doi:10.1111/anec.70235)
Supplement: Supplementary file 4 [file ANEC-31-e70235-s004.docx]

**Table S3**. Characteristics of the invasive electrophysiological study.

|  |  | Group | | |  |
| --- | --- | --- | --- | --- | --- |
|  | MBBB | | Control | |  |
| Variable | N | Mean (SD) | N | Mean (SD) | P value^*^ |
| AH Interval | 19 | 110.3 (54.0) | 17 | 102.2 (24.9) | 0.573 |
| HV Interval | 27 | 66.3 (18.3) | 18 | 52.9 (8.1) | 0.006 |

^*^Student’s t-test for independent samples; MBBB, masquerading bundle branch block; AH, Atrium-His; HV, His-Ventricle.

The comparative analysis of the means of the variables in the His bundle electrogram indicated that the mean HV interval (in ms) was higher in Group I than in Group II (66.3 ± 18.3 vs. 52.9 ± 8.1; p = 0.006) (Figure 4).
