# Supplementary material for: Maskerading Bundle Branch Block Versus Standard Bifascicular Block: A Prognostic Analysis Results Through Electrophysiological Study
Source: Ann Noninvasive Electrocardiol. 2026 Sep 12;31(5):e70235. doi: 10.1111/anec.70235 (PMC13570261; doi:10.1111/anec.70235)
Supplement: Supplementary file 5 [file ANEC-31-e70235-s005.docx]

**Figure S3. Classic bifascicular block, RBBB with S wave in lead I plus LAFB.**

Data source: Electronic health record (EHR) data
