# Supplementary material for: Maskerading Bundle Branch Block Versus Standard Bifascicular Block: A Prognostic Analysis Results Through Electrophysiological Study
Source: Ann Noninvasive Electrocardiol. 2026 Sep 12;31(5):e70235. doi: 10.1111/anec.70235 (PMC13570261; doi:10.1111/anec.70235)
Supplement: Supplementary file 6 [file ANEC-31-e70235-s006.docx]

**Table S4**. Baseline characteristics of the studied patients (Clinical parameters, variables, echocardiogram measures and EPS-HV values).

| Patient | Dyspnea | Syncope | Dec.HF | Palpitation | Gender | Age | Etiology | NYHA | ECG-rhythm | QRS duration | ECG-axis | ECHO-LA | ECHO-LVSD | ECHO-LVDD | ECHO-Septum | ECHO-PW | ECHO-EF | EPS-AH | EPS-HV | Hospitalization | Death | Pace |
| --- | --- | --- | --- | --- | --- | --- | --- | --- | --- | --- | --- | --- | --- | --- | --- | --- | --- | --- | --- | --- | --- | --- |
| **1** | **Yes** | **2** | **Yes** | **3** | **Yes** | **4** | **Yes** | **5** | **Yes** | **6** | **Yes** | **7** | **Yes** | **8** | **Yes** | **9** | **Yes** | **10** | **Yes** | **11** | **Yes** | **12** |
| **2** | **No** | **No** | **No** | **No** | **M** | **63** | **Chagas' Disease** | **I** | **Sinusal** | **130** | **-58** | **40** | **48** | **64** | **8** | **8** | **48** | **70** | **63** | **x** | **No** | **No** |
| **3** | **Yes** | **No** | **Yes** | **Yes** | **M** | **76** | **x** | **IV** | **Sinusal** | **154** | **-66** | **50** | **59** | **70** | **10** | **12** | **18** | **192** | **48** | **Yes** | **No** | **No** |
| **4** | **Yes** | **No** | **No** | **No** | **M** | **79** | **Chagas' Disease** | **II** | **x** | **x** | **x** | **46** | **72** | **80** | **8** | **7** | **21** | **x** | **70** | **x** | **Yes** | **No** |
| **5** | **Yes** | **No** | **Yes** | **No** | **M** | **72** | **Idiopathic** | **III** | **AF** | **142** | **-62** | **48** | **61** | **69** | **7** | **7** | **23** | **x** | **x** | **Yes** | **Sim** | **No** |
| **6** | **Yes** | **No** | **Yes** | **No** | **M** | **67** | **Coronary Disease** | **IV** | **AF** | **170** | **x** | **77** | **x** | **x** | **x** | **x** | **42** | **x** | **63** | **Yes** | **Yes** | **No** |
| **7** | **Yes** | **Yes** | **Yes** | **No** | **W** | **45** | **Chagas' Disease** | **III** | **AF** | **140** | **-71** | **45** | **66** | **72** | **7** | **7** | **18** | **67** | **81** | **Yes** | **Yes** | **No** |
| **8** | **Yes** | **No** | **Yes** | **No** | **M** | **73** | **Chagas' Disease** | **III** | **AF** | **182** | **-62** | **x** | **x** | **x** | **x** | **x** | **42** | **x** | **x** | **Yes** | **Yes** | **No** |
| **9** | **Yes** | **No** | **Yes** | **Yes** | **W** | **70** | **Other** | **IV** | **x** | **172** | **-64** | **50** | **57** | **66** | **8** | **8** | **28** | **174** | **105** | **Yes** | **Yes** | **Yes** |
| **10** | **No** | **Yes** | **Yes** | **No** | **M** | **73** | **Chagas' Disease** | **II** | **Sinusal** | **x** | **x** | **x** | **x** | **x** | **x** | **x** | **x** | **x** | **x** | **Yes** | **Yes** | **No** |
| **11** | **Yes** | **No** | **Yes** | **No** | **M** | **68** | **Chagas' Disease** | **II** | **Sinusal** | **158** | **-61** | **47** | **52** | **74** | **12** | **12** | **55** | **78** | **54** | **No** | **No** | **No** |
| **12** | **Yes** | **No** | **Yes** | **No** | **W** | **96** | **Coronary Disease** | **III** | **Sinusal** | **138** | **-73** | **46** | **55** | **67** | **12,5** | **9** | **36** | **132** | **60** | **No** | **No** | **No** |
| **13** | **Yes** | **Yes** | **Yes** | **No** | **W** | **49** | **Chagas' Disease** | **II** | **Sinusal** | **156** | **-60** | **42** | **49** | **60** | **7** | **7** | **37** | **78** | **80** | **No** | **No** | **Yes** |
| **14** | **Yes** | **No** | **Yes** | **Yes** | **M** | **54** | **Idiopathic** | **IV** | **Sinusal** | **166** | **-69** | **45** | **62** | **70** | **6** | **6** | **25** | **x** | **68** | **Yes** | **Yes** | **No** |
| **15** | **Yes** | **No** | **No** | **Yes** | **M** | **45** | **Idiopathic** | **II** | **AF** | **162** | **-66** | **38** | **65** | **75** | **6** | **9** | **28** | **x** | **x** | **No** | **No** | **No** |
| **16** | **Yes** | **No** | **Yes** | **No** | **W** | **71** | **Chagas' Disease** | **III** | **x** | **x** | **x** | **39** | **52** | **65** | **6** | **6** | **29** | **x** | **x** | **Yes** | **Yes** | **No** |
| **17** | **Yes** | **No** | **Yes** | **No** | **W** | **66** | **Coronary Disease** | **II** | **Sinusal** | **166** | **259** | **46** | **67** | **78** | **6** | **8** | **30** | **75** | **105** | **No** | **No** | **cdi** |
| **18** | **Yes** | **Yes** | **Yes** | **No** | **M** | **81** | **Chagas' Disease** | **III** | **AF** | **150** | **-83** | **x** | **x** | **x** | **x** | **x** | **x** | **255** | **110** | **No** | **No** | **Yes** |
| **19** | **Yes** | **Yes** | **Yes** | **No** | **W** | **65** | **Chagas' Disease** | **IV** | **x** | **x** | **x** | **40** | **59** | **71** | **8** | **8** | **34** | **93** | **60** | **No** | **No** | **No** |
| **20** | **No** | **Yes** | **No** | **No** | **W** | **70** | **Chagas' Disease** | **I** | **Sinusal** | **144** | **-62** | **40** | **41** | **57** | **8** | **8** | **54** | **170** | **38** | **No** | **No** | **No** |
| **21** | **Yes** | **No** | **Yes** | **No** | **M** | **82** | **Other** | **IV** | **AF** | **174** | **-57** | **60** | **64** | **73** | **11** | **10** | **26** | **x** | **x** | **No** | **No** | **No** |
| **22** | **No** | **No** | **Yes** | **No** | **M** | **61** | **Chagas' Disease** | **I** | **Sinusal** | **164** | **-71** | **44** | **x** | **68** | **5** | **6** | **x** | **x** | **x** | **No** | **No** | **No** |
| **23** | **Yes** | **No** | **No** | **No** | **W** | **70** | **Chagas' Disease** | **I** | **AF** | **122** | **-74** | **55** | **42** | **54** | **9** | **9** | **44** | **x** | **x** | **No** | **No** | **No** |
| **24** | **Yes** | **No** | **Yes** | **No** | **M** | **77** | **Chagas' Disease** | **III** | **Sinusal** | **152** | **-60** | **44** | **59** | **67** | **10** | **9** | **25** | **x** | **57** | **Yes** | **No** | **No** |
| **25** | **Yes** | **Yes** | **Yes** | **No** | **M** | **70** | **Chagas' Disease** | **I** | **Sinusal** | **152** | **-79** | **34** | **39** | **57** | **8** | **8** | **37** | **x** | **x** | **No** | **No** | **Yes** |
| **26** | **Yes** | **Yes** | **Yes** | **No** | **M** | **77** | **Chagas' Disease** | **III** | **Sinusal** | **176** | **-71** | **44** | **59** | **66** | **11** | **11** | **23** | **x** | **x** | **Yes** | **No** | **No** |
| **27** | **Yes** | **Yes** | **Yes** | **No** | **M** | **78** | **Chagas' Disease** | **III** | **Sinusal** | **194** | **-80** | **48** | **64** | **72** | **9** | **9** | **23** | **x** | **x** | **No** | **No** | **No** |
| **28** | **Yes** | **No** | **Yes** | **No** | **M** | **81** | **Coronary Disease** | **III** | **Sinusal** | **128** | **-77** | **41** | **51** | **60** | **13** | **13** | **32** | **x** | **57** | **Yes** | **No** | **No** |
| **29** | **No** | **Yes** | **No** | **No** | **M** | **70** | **Coronary Disease** | **I** | **Sinusal** | **152** | **-47** | **45** | **32** | **48** | **15** | **14** | **62** | **70** | **66** | **No** | **No** | **No** |
| **30** | **No** | **No** | **No** | **Yes** | **W** | **71** | **Chagas' Disease** | **I** | **Sinusal** | **142** | **-70** | **39** | **35** | **52** | **7** | **7** | **61** | **90** | **48** | **No** | **No** | **No** |
| **31** | **No** | **No** | **No** | **No** | **M** | **70** | **Idiopathic** | **I** | **x** | **x** | **x** | **50** | **45** | **57** | **9** | **9** | **42** | **x** | **x** | **No** | **No** | **No** |
| **32** | **Yes** | **No** | **Yes** | **No** | **W** | **41** | **Chagas' Disease** | **IV** | **x** | **x** | **x** | **44** | **48** | **59** | **8** | **7** | **38** | **x** | **x** | **Yes** | **No** | **No** |
| **33** | **Yes** | **No** | **Yes** | **No** | **M** | **68** | **Chagas' Disease** | **IV** | **AF** | **158** | **-20** | **50** | **57** | **64** | **8** | **8** | **23** | **x** | **x** | **Yes** | **No** | **Yes** |
| **34** | **Yes** | **Yes** | **No** | **No** | **W** | **72** | **Chagas' Disease** | **III** | **AF** | **154** | **-70** | **37** | **45** | **58** | **8** | **8** | **35** | **x** | **x** | **Yes** | **No** | **Yes** |
| **35** | **Yes** | **No** | **Yes** | **No** | **M** | **87** | **Idiopathic** | **III** | **AF** | **148** | **-61** | **53** | **62** | **80** | **11** | **11** | **44** | **x** | **63** | **No** | **No** | **No** |
| **36** | **No** | **Yes** | **No** | **No** | **M** | **70** | **Chagas' Disease** | **II** | **Sinusal** | **192** | **-88** | **x** | **x** | **x** | **x** | **x** | **x** | **x** | **x** | **No** | **Yes** | **Yes** |
| **37** | **No** | **No** | **No** | **No** | **W** | **59** | **Chagas' Disease** | **I** | **Sinusal** | **152** | **-51** | **32** | **48** | **65** | **8** | **8** | **50** | **90** | **54** | **Yes** | **No** | **No** |
| **38** | **Yes** | **No** | **Yes** | **No** | **M** | **61** | **Idiopathic** | **II** | **Sinusal** | **162** | **-85** | **37** | **54** | **64** | **6** | **11** | **32** | **x** | **x** | **No** | **Yes** | **Yes** |
| **39** | **Yes** | **No** | **Yes** | **No** | **W** | **70** | **Coronary Disease** | **IV** | **Sinusal** | **122** | **-58** | **42** | **50** | **60** | **10** | **9** | **34** | **62** | **45** | **No** | **No** | **No** |
| **40** | **Yes** | **No** | **No** | **No** | **W** | **75** | **Chagas' Disease** | **II** | **AF** | **144** | **-67** | **34** | **35** | **45** | **6** | **6** | **45** | **x** | **54** | **No** | **No** | **No** |
| **21** | **No** | **Yes** | **No** | **No** | **M** | **80** | **Coronary Disease** | **I** | **x** | **x** | **x** | **37** | **31** | **57** | **9** | **9** | **76** | **x** | **75** | **No** | **No** | **Yes** |
| **42** | **No** | **Yes** | **No** | **No** | **W** | **48** | **Chagas' Disease** | **II** | **Sinusal** | **148** | **-69** | **34** | **34** | **53** | **8** | **8** | **65** | **72** | **75** | **No** | **No** | ***Yes** |
| **43** | **Yes** | **No** | **Yes** | **No** | **M** | **65** | **Chagas' Disease** | **III** | **x** | **x** | **x** | **58** | **83** | **91** | **8** | **8** | **18** | **150** | **81** | **Yes** | **No** | **Yes** |
| **44** | **No** | **Yes** | **No** | **Yes** | **M** | **53** | **Chagas' Disease** | **II** | **Sinusal** | **152** | **-59** | **46** | **58** | **70** | **8** | **8** | **50** | **110** | **48** | **Yes** | **No** | **Yes** |
