# Supplementary material for: Maskerading Bundle Branch Block Versus Standard Bifascicular Block: A Prognostic Analysis Results Through Electrophysiological Study
Source: Ann Noninvasive Electrocardiol. 2026 Sep 12;31(5):e70235. doi: 10.1111/anec.70235 (PMC13570261; doi:10.1111/anec.70235)
Supplement: Supplementary file 7 [file ANEC-31-e70235-s007.docx]

**Table S1.** Comparison of the distribution of clinical, etiological, and electrocardiographic variables and the need for permanent pacemaker implant.

| Parameter | Group | |  |
| --- | --- | --- | --- |
|  | **MBBB** | **Control** | **P value** |
| Age (years) | N; M (SD) | N; M (SD) |  |
|  | 44; 68.0 (11.7) | 26; 68.1 (11.3) | 0.956^**^ |
| VARIABLES | **N (%)** | **N (%)** |  |
| Gender |  |  | 0.634^*^ |
| Male | 28 (63.6) | 18 (69.2) |  |
| Female | 16 (36.4) | 8 (30.8) |  |
| Syncope |  |  | 0.395^*^ |
| Yes | 16 (36.4) | 6 (26.1) |  |
| No | 28 (63.6) | 17 (73.9) |  |
| Decompensated HF |  |  | 0.053^*^ |
| Yes | 29 (65.9) | 10 (41.7) |  |
| No | 15 (34.1) | 14 (58.3) |  |
| Palpitations |  |  | **0.003^*^** |
| Yes | 7 (15.9) | 12 (50.0) |  |
| No | 37 (84.1) | 12 (50.0) |  |
| Functional class |  |  | 0.094^*^ |
| I | 10 (22.7) | 10 (43.5) |  |
| II | 11 (25.0) | 7 (30.4) |  |
| III/IV | 23 (52.3) | 6 (26.1) |  |
| Etiology |  |  | 0.733^†^ |
| Idiopathic | 5 (11.9) | 3 (13.6) |  |
| CAD | 7 (16.7) | 5 (22.7) |  |
| Chagas’ disease | 28 (66.7) | 12 (54.5) |  |
| Other | 2 (4.8) | 2 (9.1) |  |
| Pacemaker |  |  | **0.001^†^** |
| Yes | 13 (29.5) | 0 (0.0) |  |
| No | 31 (70.5) | 27 (100.0) |  |
| Death |  |  | 0.054^†^ |
| Yes | 11 (25.0) | 2 (7.4) |  |
| No | 33 (73.2) | 25 (92.6) |  |
| ECG-Rhythm |  |  | **0.018^*^** |
| Sinus l | 23 (60.5) | 22 (88.0) |  |
| Fibrillation or atrial flutter | 15 (39.5) | 3 (12.0) |  |
| ECG-Conduction-AV |  |  | 0.411^†^ |
| Normal | 26 (76.5) | 19 (82.6) |  |
| AVB First degree | 7 (20.6) | 2 (8.7) |  |
| AVB Second degree type I (Wenckebach) | 0 (0.0) | 1 (4.3) |  |
| AVB Second degree type II/advanced AVB /TAVB | 1 (2.9) | 1 (4.3) |  |
|  | M (DP) | M (DP) | **p** |
| ECG-Axis | –57.3 (55.6) | –17.3 (113.1) | 0.075^**^ |
| ECG-Duration-QRS | 154.6 (16.9) | 148.7 (19.0) | 0.211^**^ |

**Caption**: ^*^Chi-square test. ^**^Student’s t-test. ^†^ Fisher’s exact test. M: Mean; MBBB: Masquerading bundle branch block.
